# Perceptions of pharmacists towards drug shortages in the healthcare system of Pakistan and its impact on patient care: findings from a cross-sectional survey

**DOI:** 10.1136/bmjopen-2021-050196

**Published:** 2021-12-23

**Authors:** Sumaira Omer, Mengyuan Pan, Salamat Ali, Sundus Shukar, Yu Fang, Caijun Yang

**Affiliations:** 1 Department of Pharmacy Administration and Clinical Pharmacy, Xi'an Jiaotong University, Xi'an, Shaanxi, China; 2 Department of Pharmacy, Quaid-i- Azam University, Islamabad, Islamabad, Capital, Pakistan

**Keywords:** health policy, health services administration & management, general medicine (see internal medicine), quality in health care

## Abstract

**Objective:**

This study aimed to explore pharmacists’ perceptions on drug shortages and its impingement on the Pakistani healthcare system, in particular on patient care.

**Design:**

Online questionnaire survey.

**Setting and participants:**

Hospital pharmacists from five out of seven regions of Pakistan were approached; including the federal territory (Islamabad) and four provinces (Khyber Pakhtunkhwa, Balochistan, Punjab and Sindh).

**Primary and secondary outcome measures:**

Prevalence and type of shortages were identified along with strategies to reduce its effect on patient care.

**Method:**

A validated questionnaire was distributed through various online platforms to 800 registered hospital pharmacists. A convenience sampling technique was used to obtain information on drug shortages, the reporting system for shortages, the impact on patients and policy solutions for managing drug shortages.

**Results:**

Out of 800 hospital pharmacists, 708 completed the questionnaire (response rate: 88.5%). Of these hospital pharmacists, 47% came from hospitals of Punjab, 26% from Khyber Pakhtunkhwa, 13% from Sindh, 11% from Balochistan and 4% from Islamabad; 72% and 28% worked in tertiary and secondary hospitals, respectively. The majority (32%) interacted with shortages daily. The top three drug categories reported in shortage were oncology drugs (54%), cardiovascular drugs (53%) and antimicrobials (42%). 58% of the respondents have seen care delayed as a negative consequence of shortages. ‘Creating new communication system’ (65%) and ‘readjust budget plans’ (41%) were the two most frequently indicated recommendations for shortages management at hospital, while ‘circulars or alerts from the regulatory authority’ (60%) and ‘time to time directives from local health statuaries’ (48%) were two most widely suggested policy solutions.

**Conclusion:**

Drug shortage is a serious concern in Pakistani hospitals, experienced on a daily basis endangering patients’ health. Enhanced communication is required, connecting the key stakeholders. Health policies should be reviewed; adequate funds should be allocated to the health sector preventing future shortages.

**Strengths and limitations of this study**

- This was the first national quantitative study undertaken to determine the prevalence of drug shortages and its management within Pakistani hospitals.
- A modified version of the European Association of Hospital Pharmacists drug shortage questionnaire was used to obtain clear information regarding drug shortages.
- Recommendations for the management of shortages at local and national level were suggested that could help policy makers in developing strategies to overcome shortages.
- The prevalence of shortages was based on self-reported data gained from hospital pharmacists rather than official hospital reports; due to difficulties in obtaining hospital reports.
- Only hospital pharmacists took part. Physicians and nurses were excluded from this study.

## Introduction

According to the WHO, shortages of essential medicines are becoming more ubiquitous, placing additional costs on health systems and posing health risks for patients who are not receiving the drugs they need.^1^ The American Society Health-system Pharmacists defines shortage as ‘a supply issue that affects how the pharmacy prepares or dispenses a drug product or how patient care is influenced when prescribers must use alternative products’. Whereas, according to the US Food and Drug Administration, shortage means ‘a timeframe when the demand or estimated demand for the medicine excels the supply of the medicine’.^2^ Health professionals are often troubled by the stress caused by the short supply and for patients this can lead to compromised care.^3^

Medication shortages are not a new issue nevertheless it has gained momentum over recent years.^4^ A study of healthcare workers in the USA found that 99% of participants had experienced shortages in previous months prior to the study.^5^ The survey by the European Association of Hospital Pharmacists (EAHP) has similar findings: trends in shortages have increased rapidly from 2014 to 2018 in European countries. In 2014, 86% and in 2018, 92% of the participants agreed with the statement that drug shortages was an ongoing issue in terms of providing the best care or operating the hospital pharmacy.^6^

In Pakistan, the provincial government of each province is responsible for health services, including the purchase of medicines, except for regions administered by the federal government. At the federal level, the Ministry of Health plays role in the development of drug policies.^7^ Procurement procedures vary across provinces and have evolved over time. Recently, Khyber Pakhtunkhwa and Sindh (provinces) have a hybrid procurement model. In this model, the price and supplier of a medicinal product remains same for all hospitals, but the healthcare facilities purchase individually. In Balochistan (province), the Medical Store Depot centrally purchases medicines which are then transferred to public hospitals as needed. The decentralised procurement model has been used in Punjab (province) in recent years (2019).^8^ Under this system, public sector hospitals directly contact prequalified and registered pharmaceutical companies through notice and the most appropriate pharmaceutical company gets the contract.

In order to improve access to medicines in Pakistan, the National Policy on Drug Prices (NDPP) was launched in 2018. But this policy had minimal. By using WHO/HAI methodology, Amna *et al* found that the introduction of the NDPP increased the availability of brands (6.8%–33.1%), but simultaneously reduced the availability of low-priced generics (35.1%–9%) and the overall medicine availability was remained insufficient to fulfil population healthcare needs.^9^ Similarly, in Sindh (province) the availability of critical medications for acute care (30%–67%) and for long-term care (3%–57%) has been deemed insufficient. Moreover, the purchased medicines are of low quality, and there is a lack of stock management.^10^ In hospital settings, these shortages would likely impact patient safety as well as hospital performance.

Despite extensive evidence, there is limited literature on the effect of drug shortages in Pakistani hospitals. In addition, the issue has not been studied at a national level. In 2017, a study was conducted in, Karachi city, Pakistan to investigate the issues surrounding drug shortages however, this study was limited to tertiary hospitals.^11^ In total, 472 physicians and pharmacists from both public and private tertiary hospitals participated. The findings indicated that injectables were mostly subject to supply issue accounting for 52.2% of the overall shortages. It was also claimed that the shortage crisis had negative impact on patient care in terms of prolonged hospital stays and increased treatment costs. However, the study did not explore the causes of drug shortages nor its solutions.^11^

There is a lack of evidence-based research on the prevalence of drug shortages and its management within hospital setting in Pakistan and to the best of our knowledge, there are no other ongoing studies on drug shortages within secondary care hospitals in Pakistan The barriers in the management of drug shortages need to be determined and analysed to ensure optimal patient care. Therefore, this study aimed to conduct a national level survey on drug shortages targeting pharmacists of both tertiary and secondary hospitals to determine its prevalence, impact on patient care, management in the public hospital context, along with national strategies to deal with drug shortages and relevant modifications.

## Method

### Study design

An online cross-sectional survey was conducted between August 2020 and October 2020 involving registered pharmacists of public hospitals in Pakistan. Hospital pharmacists were selected as they are commonly the first health professionals to encounter drug shortages.^12^ They have the skills and knowledge to identify solutions to the problem and are well connected with manufacturers and wholesalers through their procurement and logistic roles. Moreover, they guide physicians and nurses on available treatment options.^12^

Geographically, Pakistan is a country in South Asia which comprises two autonomous territories (Azad Jammu and Kashmir, Gilgit-Baltistan), one federal territory (Islamabad) and four provinces (Khyber Pakhtunkhwa, Balochistan, Punjab and Sindh). This study included all four provinces and the federal territory. The study questionnaire was administered online to hospital pharmacists working in public secondary and tertiary care hospitals in 38 main cities (24 districts) in Pakistan. Further details are presented in online supplemental figure S1.

10.1136/bmjopen-2021-050196.supp1 Supplementary data

Online platforms were preferred to collect the data considering the fact that in Pakistan, 76 million people have internet access, and 37 million people are actively using various social media platforms.^13^

### Data collection tool

The EAHP drug shortage questionnaire 2018 was sent online to participants after a minor modification.^6^ The study questionnaire was modified by the research team through group discussions. The draft was evaluated by two experts in the pharmacy practice research for the content validity of the instrument. The questionnaire was then piloted on 50 hospital pharmacists. Detailed feedback on format, clarity and completion time was collected to make minor changes. We did not include the pilot responses in the final analysis. In total, the questionnaire (see online supplemental file S1) contained 31 questions, covering five sections that included information on participant demographics (2 questions), drug shortage information (11), medicine shortages reporting system (7), impact of shortages on patient care (6) and policy solutions for the management of drug shortages (5).

### Sampling strategy and sample size calculation

Data collection was conducted using a convenience sampling technique as there was no database present to identify the exact count of pharmacists practicing in various hospitals in Pakistan.^14^ According to the Pharmaceutical profile of Pakistan, documented in the WHO 2010 report, there were approximately 10 000 pharmacists serving in all settings of Pakistan.^15^ Therefore, the required sample size was estimated of 370 participants at 95% CI and 5% margin error. Because the nature of low response of online surveys, to ensure sample adequacy, we sent our online questionnaire to nearly 800 pharmacists who worked in the target hospitals.

The survey was conducted by the following steps: (1) participants list along with contact details for each hospital was obtained through official channels. (2) Participants were initially contacted via their official emails, WhatsApp or Facebook account. To confirm that the collected account belonged to the specific participant, a message was sent to the account to confirm his or her personal details at first. (3) After receiving the confirmation, we sent a message to each pharmacist, containing a cover letter introducing the study, the participant’s special code and a link to the online questionnaire. In this way, we send the online questionnaire to 800 hospital pharmacists.

The participants were repeatedly reminded to respond and fill the questionnaire. We sent up to five reminder messages (one reminder after 2–3 weeks) to participants, prompting them to complete the survey. An identity number was used for each participant to avoid repetition.

### Statistical analysis

The data were initially coded and entered into MS Excel, then transferred to the Statistical Package for the Social Sciences (SPSS V.20) for analysis. For categorical questions, descriptive statistics were performed using frequencies and percentages. Information on drug shortages was compared between four provinces of Pakistan and one capital territory using the χ^2^ test or the Fisher’s exact test, as appropriate. Statistical significance was determined at an alpha of 0.05. The findings were presented in percentages (frequencies). Responses to the open-ended questions were quantified and thematically analysed. A framework was developed for the qualitative analysis. Due to lack of relevant literature on the drug shortages from Pakistan, we preferred to develop a new framework for content analysis based on the concepts emerging from the data rather than using preconceived categories.^16^ Coding was performed using NVivo (software developed by QSR International for qualitative research). The initial codes were generated by tagging all common words and then were aggregated into categories. All the codes and categories were recursively reviewed by all authors and it was ensured that the generated codes and themes were truly reflective of the answers provided by the participants. Afterwards, a framework matrix was developed, with each column containing a theme and each row containing the participants’ answers. Using the coding done in the indexing phase, we read over each theme for every response provided by research participants. Next, the information was summarised and inserted into the respective cell of the matrix. Illustrative quotations were highlighted at this point. Key findings from the qualitative analysis were provided in online supplemental material S1.

Hospital pharmacists were also asked to give a numeric value for the longest duration of shortage. The longest shortage duration was estimated by finding the median duration (in months) to obtain the overall answer.

### Patient and public involvement

Patients and general public were neither involved in planning nor conducting this study.

## Results

### Characteristics of respondents

A total of 800 hospital pharmacists were approached for this study. Of those, 708 valid responses were received. There were 335 (47%) of respondents working in Punjab, 181 (26%) in Khyber Pakhtunkhwa, 91 (13%) in Sindh, 76 (11%) in Balochistan and 25 (4%) in Islamabad. The majority of the hospital pharmacists reported working in a tertiary hospital (72%), while 28% were working in a secondary hospital. The number of respondents per province is presented in table 1.

**Table 1:** Response rate of hospital pharmacists per province (n=708)

Location	Response from tertiary hospitals		Response from secondary hospitals		Total response (%)
	Total number	Average number from each tertiary hospital	Total number	Average number from each secondary hospital	
Punjab	233	12	102	5	335 (47%)
Khyber Pakhtunkhwa	148	15	33	5	181 (26%)
Sindh	70	14	21	5	91 (13%)
Balochistan	42	11	34	4	76 (11%)
Islamabad	15	15	10	5	25 (4%)
Total	508 (72%)		200 (28%)		708

### Drug shortage information

**Figure 1:**
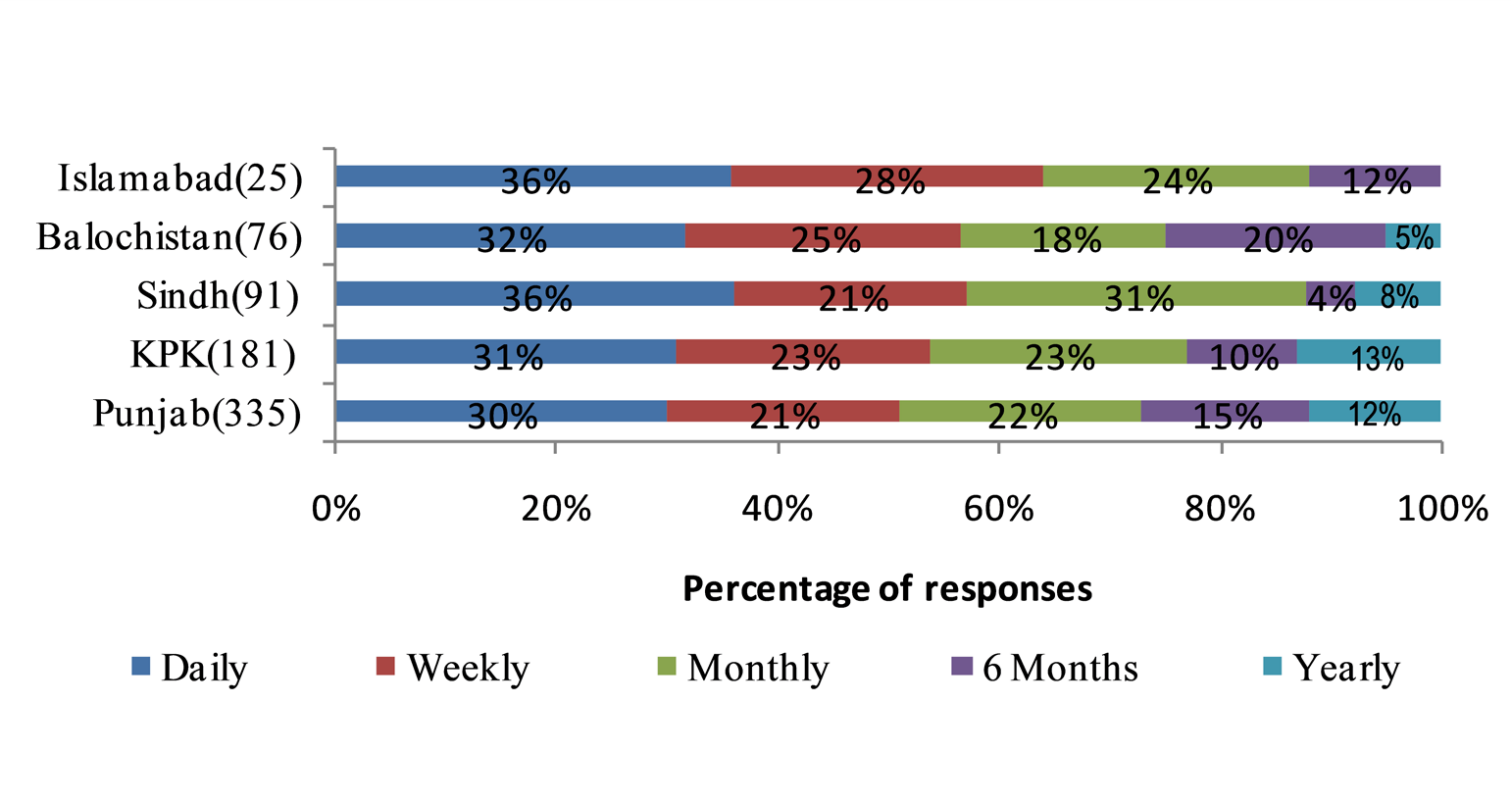
Numbers of respondents per province to the question ‘approximately how often does your hospital pharmacy experience medicine shortage?’. KPK, Khyber Pakhtunkhwa.

Among the 708 responses, 81% identified drug shortages as a current problem in providing the best care to patients. Overall, 77% of respondents indicated experiencing supply problems on a daily, weekly or monthly basis. Most respondents encountered drug shortages on daily basis in the five regions. Following this, a significantly higher percentage of pharmacists from Islamabad (28%) and Balochistan (25%) reported confronting drug shortages on a weekly basis compared with Sindh (21%), Khyber Pakhtunkhwa (23%) and Punjab (21%) as shown in figure 1.

**Figure 2:**
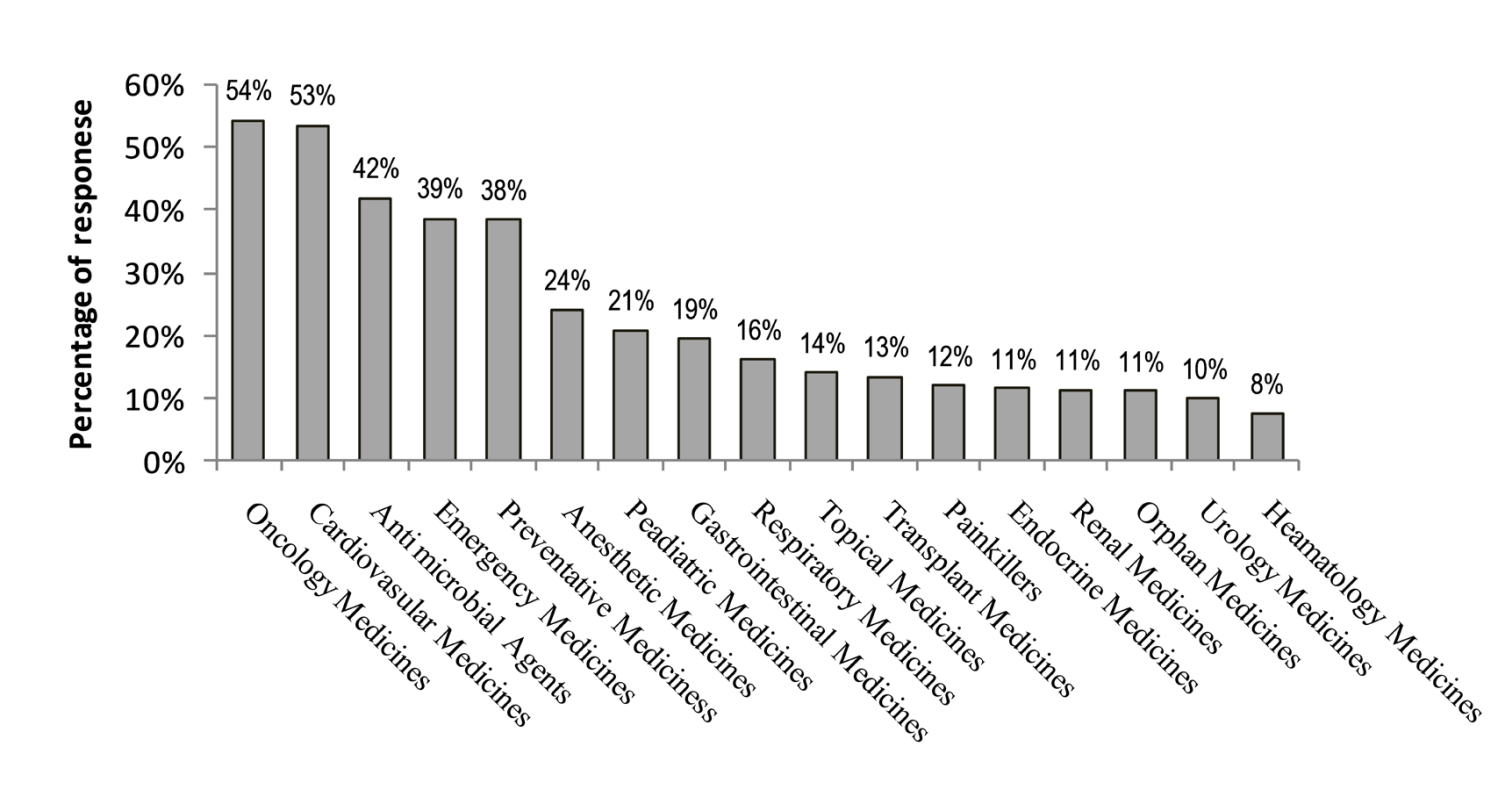
Percentage of respondents in the survey who indicated the above-mentioned categories of medicine to be frequently in short supply. (N=708). Note: this was a tick all that apply question.

Figure 2 illustrates the main categories of drugs in shortages. The top five categories of drugs were oncology drugs (54%), cardiovascular drugs (53%), and antimicrobials (42%), followed by emergency drugs (39%) and preventative drugs (38%) whereas, haematology medicines (8%) got the least response.

A list of short supplied drugs which was widely reported by the hospital pharmacists during the past years is shown in table 2.

**Table 2:** List of medicines in short supply over past months

Category	Examples of reported drugs within classes
Oncology medicine	Cyclophosphamide, etoposide, hydroxyurea, dactinomycin
Cardiovascular medicine	Isosorbide mononitrate, digoxin, glyceryl trinitrate
Antimicrobial agents	Azithromycin, cephradine, quinine, ceftriaxone, ethambutol, INH, acyclovir
Anti convulsant	Phenobarbitone, phenytoin
Preventative medicines	ASV, antirabies, TT
Anaesthetic medicines	Propofol
Nervous system agents	Tramadol, alprazolam
Anti hypertensive	Acetazolamide, verapamil, nefidipine, atropine, adenosine
Pain killer	Ibuprofen, flurbiprofen, acetaminophen, diclofenac sodium
Corticosteroids	Clobestasol, hydrocortisone
Minerals/electrolytes	Calcium gluconate
Thrombolytic drugs	SK, enoxaparin
Blood system agents	Albumin, erythropoietin
Ophthalmic drugs	Tropic amide ophthalmic solution, medicarpine drops

ASV, antisnake venom; INH, isoniazid; SK, streptokinase; TT, tetanus toxoid.

**Figure 3:**
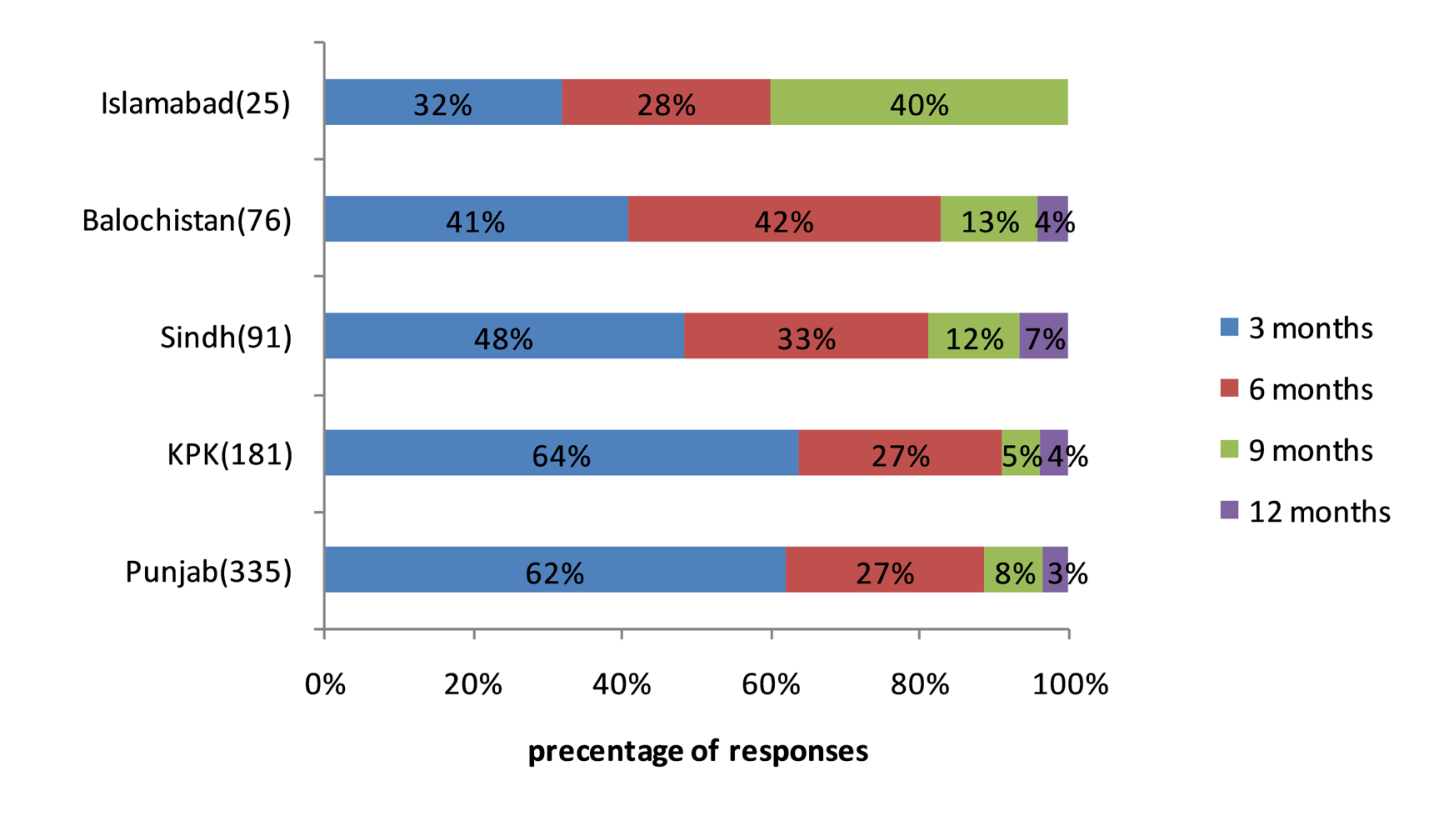
Percentage of responses to the question ‘How long would you estimate the average or typical medicine shortage normally lasts for?’ (N=708). KPK, Khyber Pakhtunkhwa.

The main drug supply sources for public hospitals in the Pakistani health system and the frequency of supply problems encountered from these sources are presented in online supplemental figure S2. Nearly 44% of the respondents claimed pharmaceutical companies as a primary supply source followed by authorised distributors (42%), local purchase vendors (8%) and ‘others category’ (6%). Purchases from pharmaceutical companies (33%) were considered less problematic than authorised distributors, followed by retailers/local purchase vendors (23%). When asked about the duration of a typical medicine shortage. The majority of Islamabad respondents’ indicated the duration of ‘9 months’. For Balochistan, ‘6 months’ (42%) duration was widely reported and nearly the same number of respondents (41%) indicated ‘3 months’. In Sindh (48%), Khyber Pakhtunkhwa (64%) and Punjab (62%) the time duration of ‘3 months’ was declared frequently. Overall, the median time duration for maximum shortage was 10 months. Figure 3 illustrates the duration of a typical shortage.

Out of 708, there were 243 valid text responses indicated personal experience with the longest shortage. This information can be found in online supplemental table S1.

### Reporting system for the short supplies

Seven questions were asked (six multiple-choice and one open-ended) to determine how information about short supplies was shared. Out of total respondents (N=708), 46% of the respondents answered ‘NO’ when asked ‘Is there any reporting system in place’. Approximately, 43% of the respondents answered ‘YES’ while 11% indicated ‘I do not know’. Details are given in table 3. The vast majority of the respondents from Punjab (50%) and Khyber Pakhtunkhwa (48%) indicated that there was a system for reporting shortages. Conversely, most respondents from Sindh (66%), Balochistan (55%) and Islamabad (88%) indicated no system exits to report medicine shortages.

**Table 3:** Drug shortage reporting system information

Information related to reporting system	Punjab	KPK	Sindh	Balochistan	Islamabad	P value	Count (%)
Is there any reporting system for shortages in place? (n=708)							
No	126 (38)	75 (41)	60 (66)	42 (55)	22 (88)	0.00	325 (46)
Yes	168 (50)	86 (48)	25 (27)	27 (36)	2 (8)		308 (43)
I do not know	41 (12)	20 (11)	6 (7)	7 (9)	1 (4)		75 (11)
What is the level of the reporting system in your hospital? (n=308)							
National level	15 (9)	4 (4)	0 (0)	1 (4)	0 (0)	0.057	20 (6)
Provincial level	57 (33)	19 (23)	8 (32)	4 (15)	0 (0)		88 (29)
Hospital level	98 (58)	61 (73)	17 (68)	22 (81)	2 (100)		200 (65)
Do you judge reporting system as effective/working/functional? (n=308)							
No	49 (29)	39 (46)	15 (60)	14 (52)	2 (100)	0.001	119 (39)
Yes	121 (71)	45 (54)	10 (40)	13 (48)	0 (0)		189 (61)
Which institution has major responsibility for management of shortages in Pakistan?							
Federal agencies	70 (21)	52 (29)	23 (25)	23 (30)	9 (36)	0.175	177 (25)
Provincial agencies	146 (44)	66 (36)	35 (39)	36 (48)	9 (36)		292 (41)
Hospital itself	119 (35)	63 (35)	33 (36)	17 (22)	7 (28)		239 (34)
Are causes for medicines shortages reported by suppliers/producers to health authorities (n=708)							
No	44 (13)	36 (20)	7 (8)	9 (12)	3 (12)	0.002	99 (14)
Yes	76 (23)	27 (15)	11 (12)	12 (16)	0 (0)		126 (18)
I do not know	61 (18)	46 (25)	27 (30)	23 (30)	6 (24)		163 (23)
Sometimes	154 (46)	72 (40)	46 (50)	32 (42)	16 (64)		320 (45)
Are causes for medicines shortages reported to hospitals? (n=708)							
No	50 (15)	32 (18)	14 (15)	14 (18)	5 (20)	0.009	115 (16)
Yes	81 (24)	40 (22)	9 (10)	12 (16)	0 (0)		142 (20)
I do not know	23 (7)	12 (7)	5 (6)	10 (13)	0 (0)		50 (7)
Sometimes	181 (54)	97 (53)	63 (69)	40 (53)	20 (80)		401 (57)

P value less or equal to 0.05 was considered significant.

KPK, Khyber Pakhtunkhwa.

In order to obtain more information on the reporting system, respondents were asked to define the level of the reporting system. This question was only asked to 308 (44%) respondents who acknowledged the existence of a reporting system. The majority of respondents indicated the reporting system of ‘hospital level’ (65%) and ‘provincial level’ (29%). Only 6% indicated a ‘national level’ reporting system (table 3). For the question ‘Which institution has the major responsibility for managing shortages’, many respondents (41%) answered ‘provincial agencies’, followed by ‘hospital itself’ (34%) and ‘federal agencies’ (25%).

Approximately, 45% respondents believed that suppliers/producers sometimes informed causes of shortages to health authorities, and more respondents (57%) declared that suppliers/producers sometimes reported to public hospitals.

There were 244 free text answers that detailed the working of reporting system. Four themes that emerged from these responses are listed in online supplemental table S2. The majority of the respondents (119) indicated that frequent shortages were communicated verbally or via written notice. Subsequent to this, 72 respondents reported that an intrainstitutional online reporting system was in place.

### Impact of shortages on patient care

In this section, respondents were asked about the competence of public hospitals to deliver patient treatment without major disruptions in a typical shortage situation. Over half of respondents (53%) declared that the shortage had a negative impact on patient care. The impact of shortages on patient care is illustrated in online supplemental figure S3. Around 58% of respondents found delayed care as a major consequence of medication shortages, accompanied by cancellations of care (30%), treatment failures (29%) and medication errors (26%). There were 61 responses in the ‘others category’

Table 4 presents widely used management strategies as well as proposed strategies to minimise the impact of drug shortages on patient care. ‘Inform prescribers about the drugs in shortage and recommend therapeutic alternatives’ was mainly used to manage short supply at the hospital level (72%), followed by ‘attempt to source the medicine from an alternative supplier’ (38%). And ‘create a new communication system’ was widely proposed strategy across all the provinces (63% from Punjab, 69% from Khyber Pakhtunkhwa, 69% from Sindh and 60% from Islamabad) except for Balochistan where majority of the respondents (62%) recommended to ‘readjusting budget plans’.

**Table 4:** Strategies used to minimise the impact of drug shortages on patient safety and care at hospital level

Strategies at hospital level	Punjab (N=335)	KPK (N=181)	Sindh (N=91)	Balochistan (N=76)	Islamabad (N=25)	P value	Total (N=708)
**How shortage is dealt in hospitals to minimise its impact on patient care? (tick all that apply**)							
Inform prescriber and recommend an alternative	232 (69)	141 (78)	67 (74)	58 (76)	14 (56)	0.078	512 (72)
Attempt to source the medicine from an alternative supplier	115 (34)	71 (39)	39 (43)	38 (50)	9 (36)	0.110	272 (38)
Investigate when the supply would restore and plan accordingly	66 (20)	42 (23)	28 (31)	21 (28)	6 (24)	0.192	163 (23)
Inform the prescriber of the shortage	56 (17)	32 (18)	12 (13)	13 (17)	2 (8)	0.691	115 (16)
Substitute (without the consultation with the prescriber/patient)	43 (13)	24 (13)	9 (10)	9 (12)	0 (0)	0.369	85 (12)
Change the formulary based on the information provided	35 (10)	25 (14)	8 (9)	15 (20)	0 (0)	0.039	83 (11.7)
Others	36 (11)	13 (7.2)	3 (3.3)	5 (6.6)	0 (0)	0.070	57 (8)
**What changes your hospital needed to deal with the shortage? (tick all that apply**)							
Create new communication system	211 (63)	124 (69)	63 (69)	44 (58)	15 (60)	0.384	457 (65)
Readjust budget plans	137 (41)	67 (37)	31 (34)	47 (62)	8 (32)	0.002	290 (41)
Re assign work and jobs	73 (22)	29 (16)	15 (17)	19 (25)	1 (4)	0.079	137 (19)
Use alternative sources other than hospital supplies	90 (27)	56 (31)	17 (19)	20 (26)	4 (16)	0.189	187 (26)
No change required	5 (1.5)	6 (3.3)	0 (0)	1 (1.3)	0 (0)	0.376	12 (2)

KPK, Khyber Pakhtunkhwa.

### Policy solutions for the management of drug shortages

Out of 708, 398 participants (56%) stated that there are legal regulations in Pakistan to ensure supply. Of these, 58% reported that the pharmaceutical industry had legally responsible for ensure supply, followed by the hospital pharmacy (30%) and wholesalers (12%). Details are provided in online supplemental table S3. The option ‘circulars or alerts from the regulatory authority’ was indicated as the best policy solution (60%) for shortage management followed by ‘time to time directives from local health statuaries’ and ‘annual reports’ with a response percentage of 48% and 30%.

In addition, there were 536 participants provided free text suggestions for shortage management, covering 11 themes. These themes are described in online supplemental table S4. The top three themes were ‘calling for the formation of clear policy related to supply problem’ (102 respondents), ‘calling for early information on shortage/automation in the supply chain’ (96) and ‘surveillance and careful monitoring of the supply chain’ (71).

## Discussion

A torrent of new studies during the last decade has explored that the drug shortage is a global problem, yet least investigated in low-income or middle-income countries, including Pakistan.^1^ This is the first national level study to document the phenomena of drug shortages in the Pakistani public hospitals. The shortage of life-saving drugs such as oncology medicine, cardiovascular medicine and antimicrobials was common. Concerning the source of supply, the majority of respondents indicated that drugs were purchased directly from manufacturers and authorised distributors were considered less desirable in this regard. Many participants reported that the health system sometimes receives advance notice of shortages. Patient suffers due to a shortage, primarily in the form of delayed care and cancelled care.

Oncology medicines (54%), cardiovascular medicines (53%) and antimicrobial agents (42%) were the three major medicine categories with a supply problem. This result is conformational to other studies wherein the drug shortage has been reported such as oncology drug shortages in Iran,^17^ Benzathine Penicillin G (antimicrobial agent) shortage in the USA,^18^ cardiovascular drugs in Saudi Arabia and China,^19 20^ antibiotics and oncology medicines shortages in Europe.^21^ Reports on the shortage of essential and lifesaving drugs were also found in the preceding years in Pakistan.^22^ The oncology drug shortage is found to be more prevalent in Pakistan because most of them are not manufactured at local level within the country.^23^

One of the causes of the shortage crisis in Pakistan is the lack of investment. A low budget is allocated to the public healthcare sector which is less than the critical threshold of $2 per capita per year recommended by the WHO.^22^ Moreover, the COVID-19 pandemic has also impacted the Pakistani healthcare system and exacerbated the shortage situation by unexpectedly increasing the demand for many drugs. Reports indicated that the utilisation of hydroxycloroquine, vitamin C and immune boosters was increased in Pakistan.^24^ However, this increased demand could not be met as a result of the excessive dependence of pharmaceutical manufacturers on imported raw materials. Pakistan imports 95% of its raw material for medicine production from other countries.^25^ Personal protective equipment was also found insufficient during the first wave of the COVID-19 pandemic.^26^

Similar to the findings of the EAHP survey (2018), the majority respondents in this study (44%) indicated that medications were purchased directly from manufacturers.^6^ In Pakistan, public hospitals procure medicines directly from manufacturers because middlemen such as wholesalers and distributors have been found to supply low-quality medicines in past. However, 20%–25% of the medicines can be purchased from the authorised distributors for the need of emergency situations.^7^

Prior information/alerts from the suppliers could possibly reduce the time spent by healthcare professionals for managing shortages. In 2013, nearly 170 shortages were managed due to the advance notification in the USA.^27 28^ In high-income countries, manufacturers are bound to provide information on the short supply to health authorities which continuously provide updates on short supply, while in many low-income or middle-income nations such reporting system is unavailable,^1^ that is why in our survey, the majority of participants (57%) indicated that the health system ‘sometimes’ received notice on short supply. Likewise, a study conducted in another developing nation, Jordan, found that the health professionals either never (31.65%) or rarely (38.38%) informed regarding the short supply and its expected duration.^29^

Our study has found that patients may experience great suffering as a result of shortages mainly in the form of delayed care (58%) and cancelled care (30%). This result is consistent with the study performed in acute hospitals of United States wherein 65% of survey participants (pharmacy directors) claimed delayed or cancelled a procedure in shortages.^30^ Delay or cancellation of the treatment is an event that can generate cascading effect on patient’s life like worsening the health condition, increasing the length of hospital stay and exposure to the communicable diseases.^31^ Delayed care during short supply was also widely reported in other previous studies.^6 32^ More seriously, in 2016, the Egyptian survey noted death was a frequent consequence on patient’s health from shortages reported by 35% of study respondents.^33^

Among the proposed strategies for shortage management at the hospital level, ‘create a new communication system to alert hospital staff’ was widely selected (65%) which was common with the study conducted in the Saudi hospitals.^19^ Over 70% of pharmacists in Saudi hospitals suggested that formation of communication tools would be a useful strategy for managing shortages.^19^ In policy solutions, respondents supported ‘circular or alerts from the regulatory authority’ (60%) and ‘time to time directives from local health statuaries’.

Following that, many respondents in comments stated that regulatory authorities have responsibility to ensure supplies. Poor regulatory control and weak penalties for law-violating pharmaceutical companies also contributed to the increasing short supply within the country.^34^ Implementing generic system of prescribing was also deemed helpful. Generic Drug Act, launched in 1972, to flourish generic prescribing in Pakistan, but it was applied until 1975 because of inflated medicines prices.^35^ For now, regulatory authority can prevent the shortage of particular brand with the implementation of revised generic prescribing policy.^7^

Active participation of healthcare professionals such as pharmacists to determine solutions for the short supply problem is also considered important, because they have direct contact with patients as well as their awareness of the significance of this problem.^21^ In our research, 64 free text responses proposed educating and training of the health professionals to tackle shortages. But there is a limited acceptance of the pharmacist role and pharmacy practice in the Pakistani healthcare setting.^36^ To counter this trend, the Canadian Pharmacists Association recommends broadening the scope of the pharmacist’s role, such as the authority to perform alternative treatment autonomously and in collaboration with prescribers.^37^ Moreover, pharmacists must develop additional skills and expertise needed to overcome shortages.

This study has few limitations, although it is the largest Pakistani study exploring the prevalence of medicine shortages and the proposed policy solutions. First, we assessed the prevalence of shortages using self-reported data obtained from hospital pharmacists rather than formal hospital reports, because currently only few hospitals officially record this data. Second, research findings may be limited as convenience sample was surveyed, and more participants from tertiary hospitals were involved. Finally, important healthcare professionals such as physicians and nurses were not included in this study. Further quantitative studies could be undertaken in the future exploring other key stakeholders’ perspectives on drug shortages.

## Conclusion

Drug shortage is a serious concern in Pakistani healthcare system, experienced daily in hospital setting, endangering patients’ health mainly in the form of delayed care. Based on our study results the health system sometimes received notice on short supplies. Therefore, early information about upcoming shortages and how long they will remain is seen as important measure to help manage the problem, with that enhanced communication is also required, connecting the key stakeholders. Health policies should be reviewed and more funds should be allocated to the health sector preventing shortages further escalation.

## Data Availability

Data are available upon reasonable request. Survey data sets will be provided upon receiving reasonable request. Please contact Caijun Yang: yangcj@xjtu.edu.cn for data requests.
