# Supplementary material for: Perceptions of pharmacists towards drug shortages in the healthcare system of Pakistan and its impact on patient care: findings from a cross-sectional survey
Source: BMJ Open. 2021 Dec 23;11(12):e050196. doi: 10.1136/bmjopen-2021-050196 (PMC8713015; doi:10.1136/bmjopen-2021-050196)
Supplement: Supplementary data [file bmjopen-2021-050196supp001.pdf]

Supplementary S1

Dear Participant Our study aim to collect data on drug shortages and its impact on health care system of Pakistan in particular on patients. Being a hospital pharmacist, you are most familiar with drug shortages. Your cooperation is critical to accomplish this project. We requesting your support for this study, we assure you that any information related to your identification will be kept strictly confidential and will not be disclosed at any stage of the study.

To become part of this study as a respondent, you agreed that

1. You have read the information given above;
2. You voluntarily agree to be part of this research and provide necessary information.

Q1. Hospital location:

- Provinces: _______ City:_______ Districts/Counties _______

Q2.Please chose the type of hospital/institution which you are currently working?

- Secondary care hospital
- Tertiary care hospital

Q3. Are shortages of medicines a current problem in the hospital you work in, in terms of delivering the best care to patients and/or operating the hospital pharmacy?

- Yes
- No
- Unsure

Q4. Approximately how often does your hospital pharmacy experience medicines shortages?

- Daily
- Weekly
- Monthly
- 6 Months
- Yearly
- Occasionally(Once in more than one year)

Q5. Which type of medicine do you most commonly experience to be in short supply?

- Branded medicine
- Generic medicines
- Bio similar medicines

Supplementary S1

Q6. In which area of medicine does your hospital experience shortage most commonly? Tick all that apply.

- Antimicrobial agents
- Preventative medicine
- Oncology medicine
- Anesthetic medicines
- Cardiovascular medicines
- Emergency medicine
- Hematology medicines
- GIT medicines
- Topical treatments
- Endocrine medicines
- Pediatric medicines
- Respiratory medicines
- Urology medicines
- Orphan medicines
- Renal medicine
- Transplant medicines
- Pain killers

Q7. Please tell us the medicines in which the experience of shortages was most frequent during the past year. If possible, please include their brand name, INN (International non-proprietary name) and common indications as appropriate or available? (Open ended Question)

Q8. Which of the below categories best describes the main external source of supply of medicines to your hospital?

- Direct from the Pharmaceutical company
- Authorized Distributor
- Retailers
- Others, please specify_______________

Q9. From which category of external supply does your hospital most frequently encounter problems with sourcing specific required medicines?

- Direct from the Pharmaceutical company
- Authorized Distributor

Supplementary S1

- Retailer
- Others, please specify______________

Q10. How long would you estimate the average or typical medicine shortage normally lasts for?

- 3 months
- 6 months
- 9 months
- 12 months

Q11. Please provide details about your personal experiences with typical shortage situations?

- Policy adjustment
- Drug by alternative source
- Substitution Of the drug
- Others, please specify

Q12. What is the longest duration that you can recall a medicine being in shortage for in the last 4 years? Please provide numerical values in years/months/days (open ended question)

Q13. Please provide details about the longest shortage you have personal experience, including the name and the reason for that particular shortage.

Q14. Is there any reporting system for shortages in place?

- Yes
- No
- I don't know

Q15. What is the level of the reporting system in your hospital?

- National level
- Provincial level
- Hospital level

Supplementary S1

Q16. Please explain how reporting system works and include website if available?(Open ended question)

Q17. Do you judge it as effective/working/functional?

- Yes
- No

Q18.Which institution has a major responsibility for management of shortages in Pakistan ?

- Federal Agencies
- Provincial Agencies
- Hospital itself

Q19. Are causes for medicines shortages reported by suppliers/producers to health authorities?

- Yes
- Sometimes
- No
- I don’t know

Q20. Are causes for medicines shortages reported by suppliers/producers to hospitals in Pakistan?

- Yes
- Sometimes
- No
- I don’t know

Q21. In the case of a medicine in short supply, how often do you estimate your hospital is able to provide treatment to a patient by providing a therapeutic equivalent or near equivalent medicine, without major disruption to their treatment?

- Never
- Rarely
- Sometimes
- Most of the time

Supplementary S1

- All of the time

Q22. Do you agree with the following statement? "Medicines shortages in my hospital are having a negative impact on patient care."

- Strongly disagree
- Disagree
- Unsure
- Agree
- Strongly agree

Q23. Please provide any relevant examples from your hospital over the past year of the impact medicines shortages have had on patient safety welfare. Tick all that apply.

- Delay of care
- Cancellations of care
- Medication error
- Suboptimal treatment
- Increased length of hospital stay
- Treatment failure
- Transfer of a patient to a facility where a medicine can be provided
- Adverse events
- Readmission due to treatment failure
- Death
- Others (please specify)

Q24. Please describe practically how a medicine in short supply is usually dealt with to minimize the impact on patient care. (Please tick all that apply)

- Inform prescriber and recommend an alternative
- Attempt to source the medicine from an alternative supplier
- Investigate when the supply would restore and plan accordingly
- Inform the prescriber of the shortage
- Substitute (without the consultation with the prescriber/ patient)
- Change the formulary based on the information provided
- Others(please specify)

Supplementary S1

Q25. What changes (if any) in practice has your hospital needed to make in order to deal with the shortage problem? (Please tick all that apply)

- Create new communication system to alert hospital staff regarding shortages
- Readjust budget plans because of the increased expenditure caused by shortages
- Reassign work and job descriptions because some staff have to deal with shortage specifically
- Use alternative sources other than hospital supplies
- No change required
- Others, please (specify) ______________

Q26. Please provide any relevant anecdotal evidence from your hospital over the past year of the impact medicines shortages have had on patient safety and welfare? (Open ended question)

Q27. Are there any legal regulations in Pakistan to ensure supply over a certain period of time?

- Yes
- No
- I don't know

Q28. For whom? (Are there any legal regulations in Pakistan to ensure supply over a certain period of time?) (Tick all that apply)

- Hospital pharmacy
- Wholesaler
- Pharmaceutical Industry

Q29. Are there any legal requirements in Pakistan to ensure medicine supplies over a certain period of time?)

- One month
- Two months
- Three months
- Longer (please specify)__________

Q30. Which of the following proposed policy solutions on medicines shortages would you personally support? (Tick all that apply)

- Circulars or alerts from the regulatory authority
- Time to time directives from local health statuaries

Supplementary S1

- Annual reports
- Others(please specify)______________

Q31. Please make any further comments that might be helpful to us in policy and advocacy activity on the topic of medicines shortages, including reflections on causation, reports of impacts, proposed solutions, and sources of evidence.

Supplementary S1

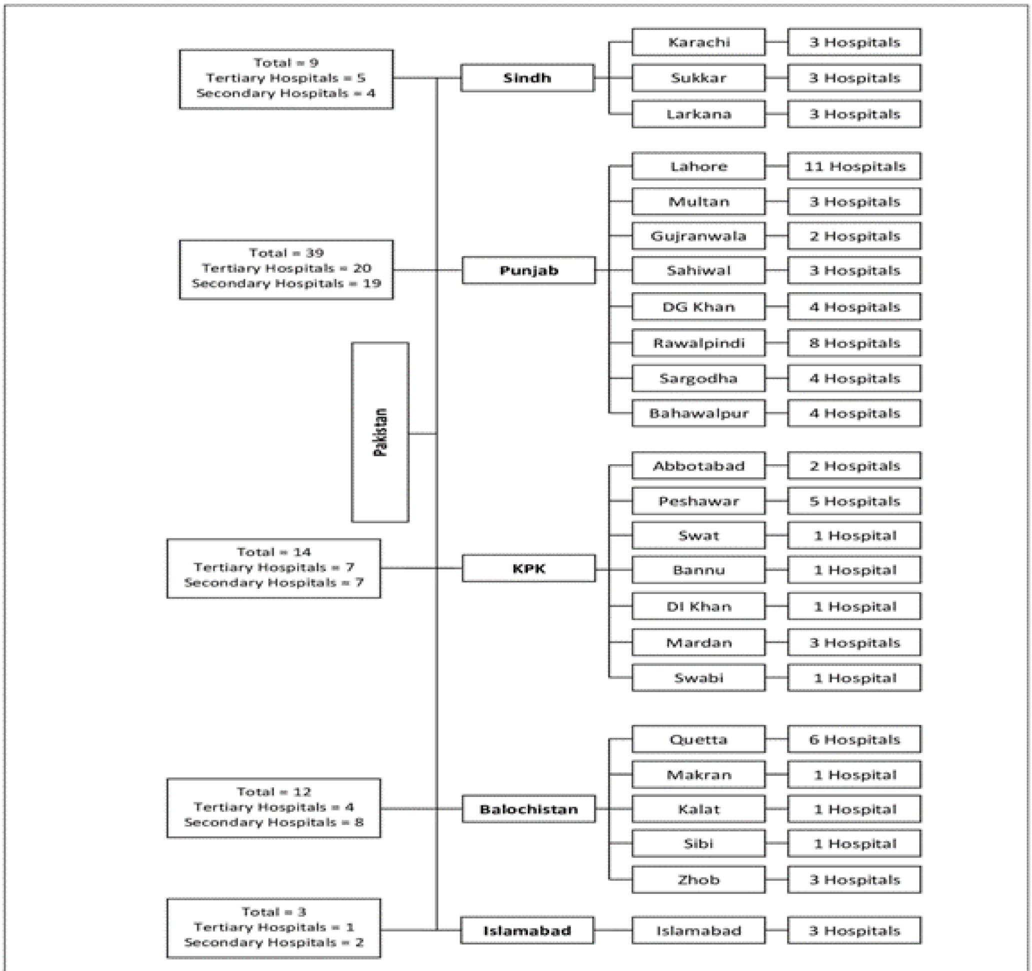

Fig. 1 Study venues

Figure1. Study Venue

Supplementary S1

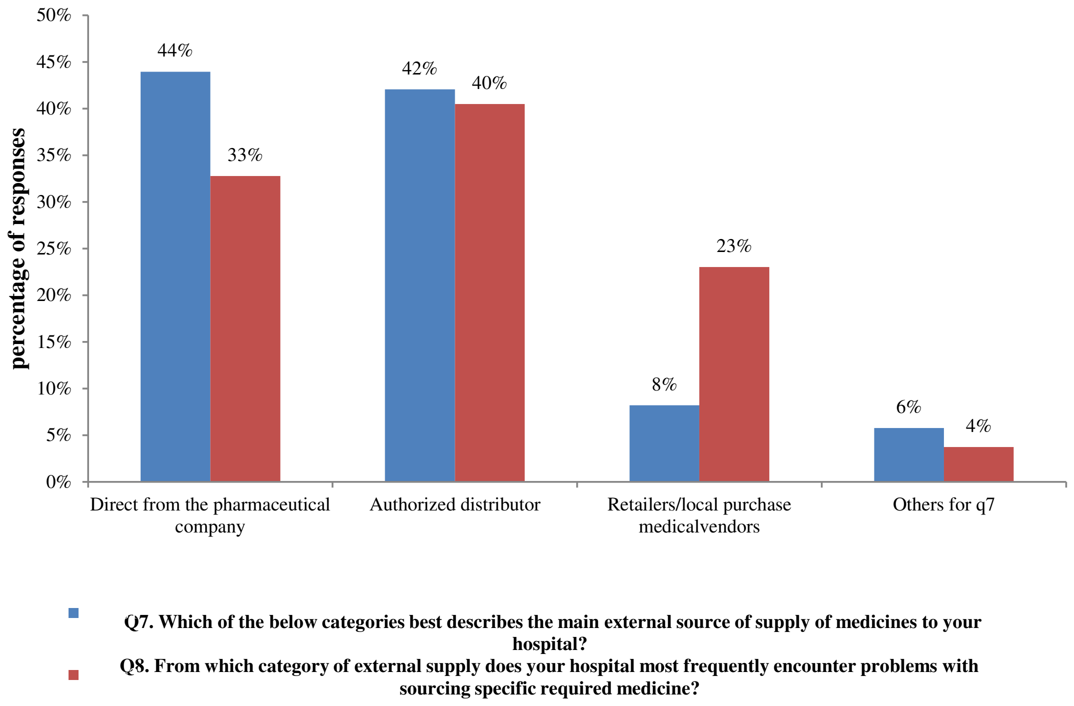

Figure2. Percentage of responses where this source of supply is identified in questions 7 and 8 of the survey

Supplementary S1

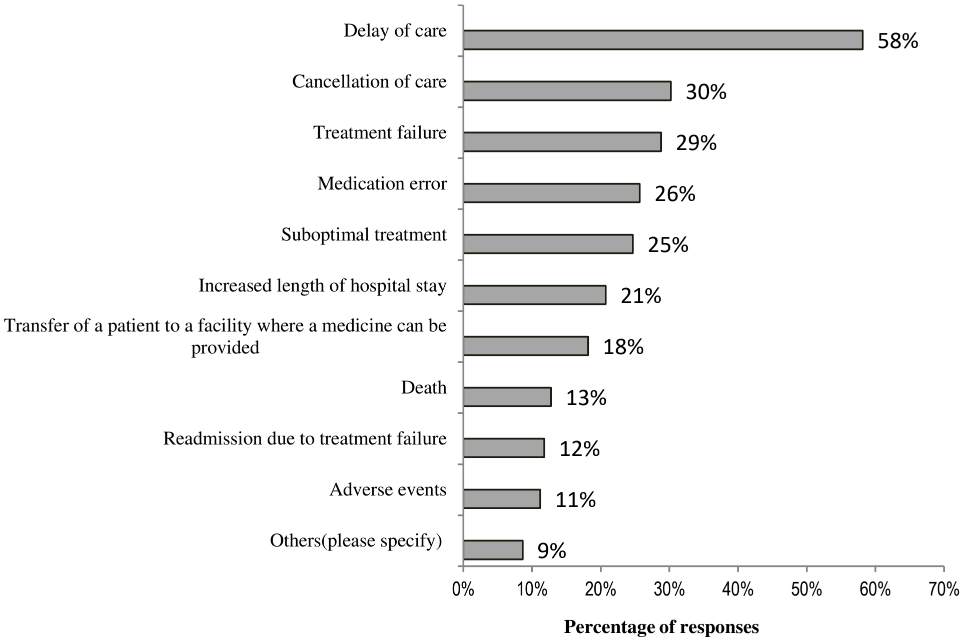

Figure3. Impact of the medicine shortage on patient care (N=708) (Note that this was a tick all that apply question)

Supplementary S1

Table s1. Detail information about the longest shortage

Themes	Examples	Number of statements
Up to 6 months	*“I have experienced a longest shortage of Ceftriaxone for 6 months”*	90
	“About 6 months of Nootropil shortage”.	
	“Antibiotic Piperacillin/Tazobactum was not available for about 6 months. Although there were therapeutic alternative available, it posed a great deal of inconvenience for patient care”	
More than 6 months to1 year	*“Dactinomycin and MESNA for 8 months”*	57
	“Shortage of Ativan for 12 months”	
	“1 year such as diamox	
For a period of two years	*“Carbamezipene for 1-2 year”*	27
	“Perfectil (approx 1.5 years)”	
	“Immunoglobulin’s was in short supply for 2 years”	
For a period of 3 years	*“Enoxaparin shortage more than 2 years”*	9
	Tab Urixin shortage for 3 years	
	Controlled drugs for 3 years	
Over 3 years	*3 to 4 year shortage of Acetazolamide*	60
	Hydrocortisone because pharmaceutical company did not supply for many years	
	I have experienced the longest shortage of plasma substitutes in a hospital that is from 2016 to till date.	
Total number of respondents		243

Abbreviations: MESNA; Sodium 2-mercaptoethane sulfonate

Supplementary S1

Table s2. participants' explanation about how the reporting system works

Themes	Examples	Number of statements
Verbal reporting, or written notice of short supplies to regulatory agencies	*"No online reporting system only verbally reporting has done or at a maximum of notifying through written notice."*<br><br>*"A complaint is registered through letters to higher authorities to address the issue of a shortage of medicines."*<br><br>*"No specific website in my knowledge, but it is mostly conveyed to the procurement department of the government or we should specifically write to the regulatory agencies."*	119
Intra institutional online reporting system	*"Internal hospital reporting system which does not have any website."*<br><br>*"Medicine shortage is only reported internally through software, not on websites."*<br><br>*"It was only designed to tell hospital staff of any sudden shortage".*	72
Record for drug shortages in inventory system but no reporting system/platform for drug shortages	*"Not reporting system available, item list is checked by our management on daily basis"*<br>*"Only have inventory system from where we find the short supplies"*<br><br>*"Record at hospital level but no proper reporting system"*	30
Centralized reporting system/online platforms for registration of complaints related to shortages	*"Official website of Drug Regulatory Authority Of Pakistan. It notifies shortage alert (www.dra.gov.pk)."*<br><br>*"mims.pshealthpunjab.gov.pk". Medicine inventory management system which is centralized electronic system has comprehensive stock information about medicines across all public districts and tehsil Headquarters hospitals within Punjab but it does not provide a solution to short supplies.*<br><br>*"There is a "WhatsApp group" with pharmacists and officers from medical depot stock control so; we can share our problems there."*	23
Total number of respondents		244

Supplementary S1

Table s3. Proposed policy solutions for drug shortages by hospital Pharmacists

**Proposed Policy Solutions**	**Punjab (335)**	**KPK (181)**	**Sindh (91)**	**Balochistan (76)**	**Islamabad (25)**	**P value**	**Total N=708**
Which of the following proposed policy solutions to shortage you personally support (tick that all apply?)							
Circulars or alerts from the regulatory authority	187(56)	121(67)	61(67)	46(61)	10(40)	.017	425(60)
Time to time directives from local health statuaries	151(45)	86(48)	43(47)	55(72)	7(28)	.000	342(48)
Annual reports	85(25)	67(37)	24(26)	32(42)	2(8)	.001	210(30)
Others	28(8.4)	28(16)	13(14)	24(32)	8(32)	.000	101(14)
No answer	7(2.1)	0(0)	0(0)	0(0)	0(0)		7(1)

Abbreviations: KPK; Khyber Pakhtunkhwa

Table s4. Participants' comments on policy and advocacy activity of the medicine shortages, including reflections on causation, reports of impacts, proposed solutions, and sources of evidence.

**Themes**	**Examples**	**Number of statements**
Calling for formation of clear policy related to supply problem	*"More effective policy is needed now as drug shortages are increasing day after day."* *"A rethink of policy is the need of the hour"* *"A policy should be formed that focus on penalized for stock piling and effective management should be developed at every level to deal with supply interruptions"*	102
Wanting early information on shortage / automation in the supply chain	*"Hospitals should be in direct contact with the manufacturer. For this, some centralized and easily accessible system must be designed to report the shortage. Where it should be addressed on time."* *"Estimated duration of shortage should be communicated on time so that alternative treatment could be provided for that time period".* *"One of the useful strategies I consider would be reporting of short supply on time, so possible alternatives can be searched to avoid the harmful consequences before the shortage".*	96
Wanting surveillance and careful monitoring of the supply chain	*"Check on medicine use after every 6 months and estimate the availability of drugs may reduce the shortage of medicine."* *"There must be accurate and active checks so that the bribery system can be avoided."* *"There is an urgent need for an audit to estimate the local pharmaceutical reserves in the country and a list of essential medicines should be revised."*	71
Wanting regulatory agencies to ensure supplies	*"Higher authorities should play their role to manage short supply".* *"Authorities should make sure the availability of life saving medicines for poor patients in Public hospitals."*	56

Supplementary S1

	"The manufacturing of some products have been stymied by restrictions on contract manufacturing and, as per the law, until those restrictions are removed the products cannot be manufactured so regulatory authority should work on this part."	
Wanting an effective strategy for drug procurement	*"System of procurement should be changed and pharmacists must be involved in this process."* *"Medicine should be provided according to the demand and make sure the presence of alternative brands with the same indications and also educate pharmacists regarding drug procurement."* *"Hospital pharmacists should carefully does drug procurement."*	36
Educating and training of the health professionals to tackle shortage and flourish generic Prescribing	*"Medicine related issues should be tackled by the pharmacist. They should get proper training for it"* *"I feel that our health system does not fulfill the needs of the society. We should take steps to uplift and promote the health system by training of pharmacist and other health care professionals"* *"One way to reduce the drug shortage is to promote generic prescribing."*	64
Low health care Budget	*"The system would be improved automatically when timely payments and enough budgets would be given to the hospital by the government. Vendors will give medicines of good brands and quality."* *"Government should increase the hospital budget."* *"In Pakistan, public hospitals are facing a big problem in constant medicine supply due to many reasons, the top of which is the poor budgeting to government hospitals."*	26
Calling for Hiring staff at hospital level	*"Pharmacists should be appointed in the administration department of hospitals because they know the medicine and its regulation."* *"Hire proper staff having duties specified in tackling the issue of drug shortage and they must be fully responsible to ensure timely supply of all the essential medicines."* *"Staff with the expertise to manage shortage should be appointed in hospitals."*	25
Wanting suppliers /manufacturers to be regulated and motivated for maintaining a continuous supply of drugs	*"All pharmaceutical industries and the authorized distributors should supply the medicine in the specified time period."* *"Pharma industries should be bound to deliver the drugs on time."* *"There should be a greater clarity on the responsibility of pharmaceutical manufacturers."*	29
Investigation on the main reason for the shortages	*"Regulatory agencies should start investigating the root causes of drug shortages and take preventive actions accordingly."* *"The long-term drug registration process is one of the reasons drug shortages occur, so first identify those reasons and take action accordingly."* *"Drug shortage is a complicated phenomenon for numerous reasons. More and more work should be done to identify these*	21

Supplementary S1

	underlying reasons."	
Calling for importation of drugs	*"A system should be introduced in which a statutory regulatory order is issued to allow patients or hospitals to import medicines to overcome shortages."* *"To reduce shortages, drugs could be imported from neighboring countries."* *"Importing drugs may be useful in meeting patient health needs."*	10
	Total number of respondents	536
